# Protocol for the production and reconstitution of VDAC1 for functional assays

**DOI:** 10.1016/j.xpro.2024.103240

**Published:** 2024-08-09

**Authors:** Grace I. Dearden, Varun Ravishankar, Ken-taro Sakata, Anant K. Menon, Lucie Bergdoll

**Affiliations:** 1 Department of Biochemistry, Weill Cornell Medical College, New York, NY 10065, USA; 2 Laboratoire d'Ingénierie des Systèmes Macromoléculaires, CNRS - Aix Marseille Université, 31 Chemin Joseph Aiguier, 13402 Marseille, France

**Keywords:** Single-molecule Assays, Metabolism, Protein Biochemistry, Protein expression and purification

## Abstract

The voltage-dependent anion channel (VDAC) is an abundant and multifunctional outer mitochondrial membrane protein, playing key roles in neurodegeneration, apoptosis, and mitochondrial membrane biogenesis. Here, we present a protocol to produce and reconstitute high yields of detergent-solubilized VDAC, expressed as inclusion bodies in *E. coli*. We describe steps for purification by affinity chromatography and refolding in lauryldimethylamine-N-oxide (LDAO). We then detail procedures for reconstituting VDAC into membrane vesicles to assay its channel and phospholipid scramblase activity via fluorescence-based assays.

For complete details on the use and execution of this protocol, please refer to Bergdoll et al.,^1^ Queralt-Martín et al., ^2^ and Jahn et al.^3^

## Highlights

• Expression of VDAC1 in *E. coli* and purification in urea
• Detailed description of protein folding in LDAO
• Step-by-step details of VDAC1 cross-linking and reconstitution into liposomes
• Fluorescence-based assay of VDAC channel and scramblase activity

## Abstract (editor highlights)

Publisher’s note: Undertaking any experimental protocol requires adherence to local institutional guidelines for laboratory safety and ethics.

## Graphical abstract

**Figure:**
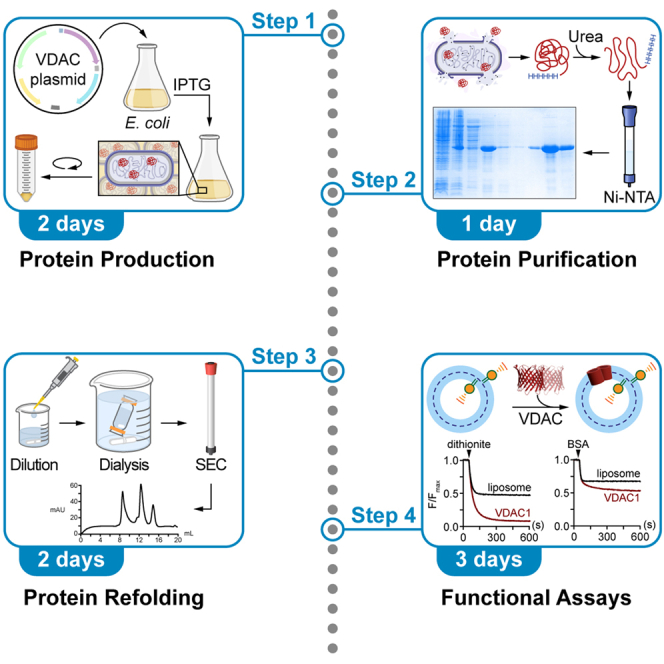

## Before you begin

The voltage-dependent anion channel (VDAC) is the most abundant protein in the outer mitochondrial membrane and plays many roles in mitochondrial physiology, ranging from ion and metabolite permeation to apoptosis and phospholipid scrambling. To study its many functions *in vitro*, purified protein is needed. Overexpression of VDAC in eukaryotic cells is challenging as it results in poor yields, and purification from tissue does not allow separation of the different isoforms. Herein, we describe a bacterial expression protocol to obtain high yields of pure and functional VDAC1, suitable for functional and structural studies. This protocol typically increases yield by 1.5- to 2-fold over protocols that rely entirely on dialysis during refolding, with some batch-to-batch variation of about 10%–20%.

**Figure 1:**
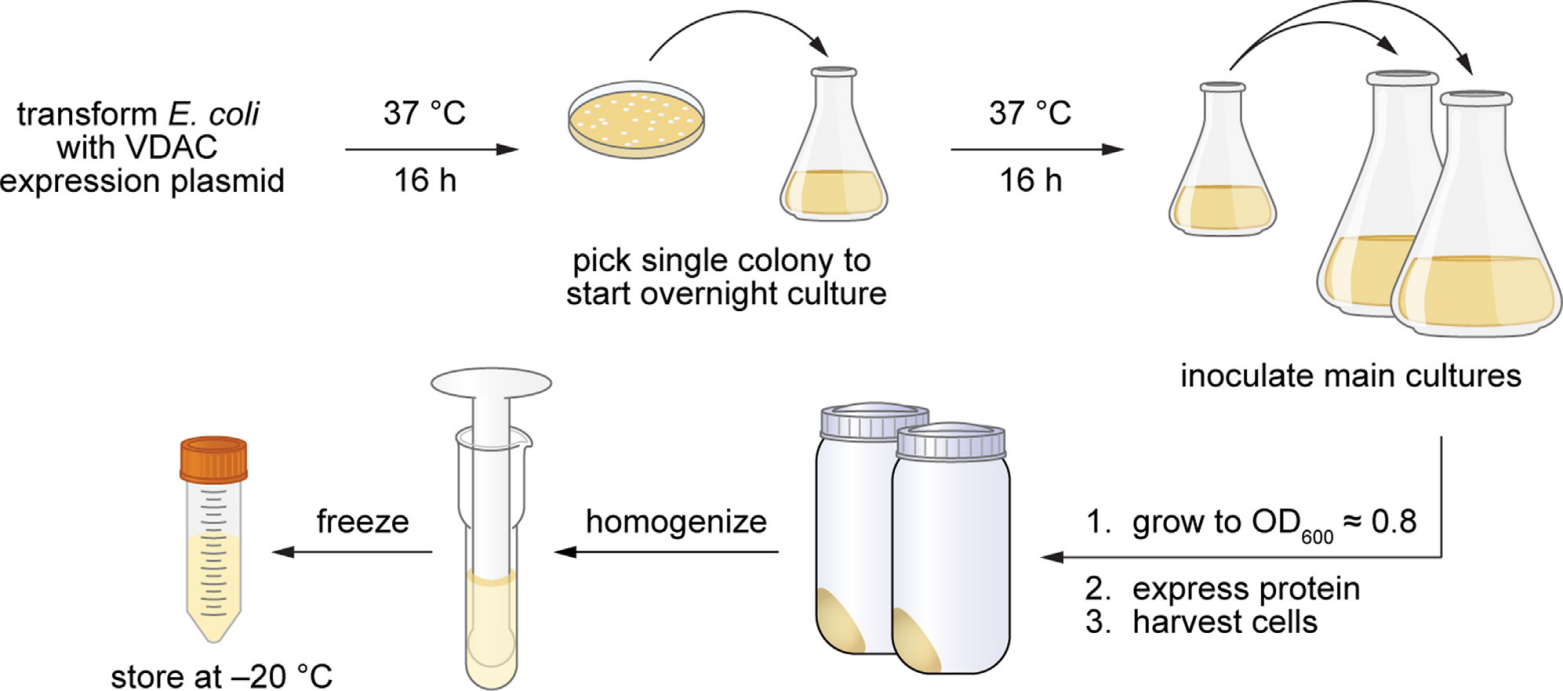
Workflow for expression of recombinant VDAC1 in E. coli Expression starts with inoculation of an “overnight culture” with a single colony of *E. coli* carrying a 6xHis-mVDAC1 or 6xHis-hVDAC1 expression plasmid. After induction with IPTG, cells are harvested and can be stored at −20°C.

**Figure 2:**
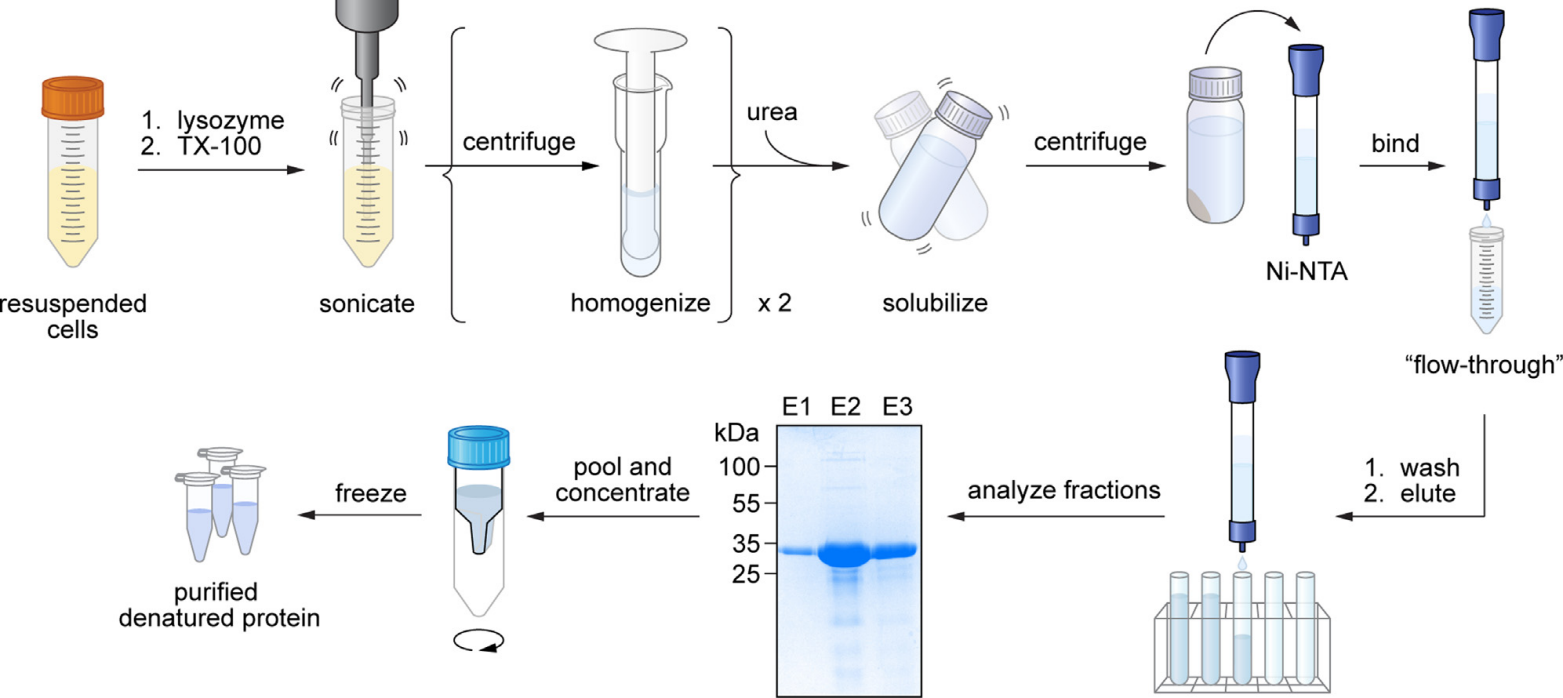
Workflow for purification of VDAC1 inclusion bodies from *E. coli* Purification starts with chemical and mechanical lysis of *E. coli* that have expressed VDAC1 as inclusion bodies. Inclusion bodies are solubilized in urea and purified by affinity chromatography. Aliquots of purified protein can be stored at −20°C.

We detail the production of 6xHis-tagged VDAC1. Mouse and human VDAC1 (mVDAC1 and hVDAC1, respectively) are essentially identical proteins, differing in only 4 amino acids, and their structure is almost identical. Consequently, the protocol we describe can be used to produce and purify both proteins and is also readily applicable to point mutants of these proteins. We express VDAC1 in *E. coli* where it forms cytoplasmic aggregates called inclusion bodies (Figure 1). The cells are lysed to release the aggregates, which are then denatured and solubilized in urea, before being subjected to Ni-NTA affinity chromatography to purify the protein (Figure 2). Purified VDAC1 is refolded by removing urea and adding detergent. Size exclusion chromatography is used as a final polishing step to eliminate residual urea and any aggregated or multimerized protein (Figure 4). 1 L of bacterial cell culture typically yields 5 mg of refolded monomeric VDAC1.

We describe procedures to assess VDAC1 channel and phospholipid scramblase activity via fluorescence-based assays (Figure 7). Channel activity does not depend on the quaternary structure of VDAC1, but scramblase activity requires VDAC1 dimers. We describe the preparation of these dimers by chemical crosslinking and a protocol to reconstitute them into large unilamellar membrane vesicles containing a trace amount of a fluorescent phospholipid reporter. Channel activity is revealed by adding the membrane-impermeant bleaching agent sodium dithionite; fluorescent lipids in the inner leaflet can only be bleached if the vesicle contains a VDAC1 channel. Scramblase activity is assayed by extracting fluorescent phospholipids from the outer leaflet of the vesicles with bovine serum albumin, resulting in the quenching of the fluorescence signal. Fluorescent lipids in the inner leaflet of vesicles can only be captured by albumin if they are scrambled to the outer leaflet by functional, scramblase-active VDAC1 dimers.

### Prepare antibiotic selection plates

**Timing: 2 h**

In this protocol, we express 6xHis-VDAC1 in *E. coli* cells. The human VDAC1 (hVDAC1) construct is encoded in the pCold plasmid which confers ampicillin resistance and can be expressed in BL21 (DE3) cells. The mouse VDAC1 (mVDAC1) construct is encoded in the pQE9 plasmid, which uses the T5 promoter and requires expression in M15 pREP4 *E. coli* cells. Because pQE9 confers ampicillin resistance and pREP4 confers kanamycin resistance, growth media must always contain both selective drugs when preparing mVDAC1.

Human and mouse VDAC used in this protocol are expressed from different plasmids for purely historical reasons. The human construct could be cloned into pQE9, or the mouse construct into pCold, without any anticipated effect on the purification protocol.

1. Add 20 LB agar medium capsules, 500 mL ddH_2_O, and a large stir bar to a 1 L Erlenmeyer flask and autoclave at 121°C for 15 min.
2. Stir the hot LB agar solution until it has cooled somewhat but is still a liquid. You should be able to hold the warm flask in your hand for 5 s.
3. Using sterile technique, add antibiotics to the warm LB agar and stir to combine.
  a. pCold 6xHis-hVDAC1: add 500 μL 100 mg/mL ampicillin (final 100 μg/mL ampicillin).
  b. pQE9 6xHis-mVDAC1: add 500 μL 100 mg/mL ampicillin and 500 μL 50 mg/mL kanamycin (final 100 μg/mL ampicillin and 50 μg/mL kanamycin).
4. Using sterile technique, pour ∼25 mL LB agar into each sterile petri dish. Cover the dish and let the agar set on the benchtop.
5. Store plates at 4°C for several months.

***Note:*** 100 mg/mL ampicillin and 50 mg/mL kanamycin stock solutions should be prepared in ddH_2_O, filter sterilized through a 0.2 μm syringe filter, and stored at −20°C in 500 μL aliquots. The frozen stocks are stable for several months.

***Note:*** Sterile technique should be used when preparing and handling bacterial strains, media, and plates. Wipe down benches and equipment with 70% ethanol. Sterilize inoculating loops and flask rims with a Bunsen burner flame.

### Transform *E. coli* with VDAC1 expression plasmid

**Timing: 2 h**

6. Obtain pCold 6xHis-hVDAC1 plasmid DNA from Anant Menon or pQE9 6xHis-mVDAC1 plasmid DNA from Lucie Bergdoll.
7. Thaw 25–50 μL competent cells (BL21 for pCold expression, M15 pREP4 for pQE9) on ice. Add 10–100 ng of plasmid and very gently tap or swirl the tube to mix.
8. Incubate on ice for 30 min.
9. Heatshock by submerging the tube in a 42°C water bath for 15 s (BL21) or 60 s (M15 pREP4).
10. Place the cells back on ice for 5 min.
11. Add 500 μL LB medium and grow for 1 h at 37°C, 200 rpm.
12. Plate 50 μL of the transformed cells on LB agar plates containing the proper selective drugs.
  a. pCold 6xHis-hVDAC1: 100 μg/mL ampicillin.
  b. pQE9 6xHis-mVDAC1: 100 μg/mL ampicillin and 50 μg/mL kanamycin.
13. Incubate 16–20 h at 37°C.

## Key resources table

**Table:** 

REAGENT or RESOURCE	SOURCE	IDENTIFIER
**Antibodies**		

Mouse monoclonal anti-6x-His (clone HIS.H8), dilution 1/1000	Thermo Fisher Scientific	Cat#MA1-21315; RRID: AB_557403

**Bacterial and virus strains**		

*Escherichia coli*: M15 (pREP4) competent cells	Creative Biolabs	Cat#MOFY-0822-FY509
*Escherichia coli*: BL21 (DE3) competent cells	Lucigen (LGC, Biosearch Technologies)	Cat#60401

**Chemicals, peptides, and recombinant proteins**		

Ampicillin	Gold Biotechnology	Cat#A-301-5; Cas: 69-52-3
Kanamycin	Sigma-Aldrich	Cat#K4000; Cas: 25389-94-0
LB agar medium capsules	MP Biomedicals	Cat#3002231
LB Medium	Sigma-Aldrich	Cat#71753-M
IPTG (isopropyl β-D-1-thiogalactopyranoside)	Sigma-Aldrich	Cat#420322; Cas: 367-93-1
Tris Base	Fisher Scientific	Cat#02-004-506; Cas: 77-86-1
HCl, 37% solution in water	Thermo Fisher Scientific	Cat#42379-5000; Cas: 7647-01-0
EDTA	Sigma-Aldrich	Cat#EDS; Cas: 60-00-4
Sucrose	Sigma-Aldrich	Cat#S9378; Cas: 57-50-1
CaCl_2_	Thermo Fisher Scientific	Cat#C79; Cas: 10035-04-8
NaCl	Sigma-Aldrich	Cat#746398; Cas: 7647-14-5
Lysozyme	Sigma-Aldrich	Cat#L2879; Cas: 9066-59-5
Triton X-100	Anatrace	Cat#T1001; Cas: 9002-93-1
Urea	Sigma-Aldrich	Cat#U1250; Cas: 57-13-6
Imidazole	Sigma-Aldrich	Cat#I5513; Cas: 288-32-4
DTT (dithiothreitol)	Thermo Fisher Scientific	Cat#R0861; Cas: 3483-12-3
LDAO (Lauryldimethylamine-N-Oxide)	Anatrace	Cat#D360S; Cas: 1643-20-5
Ni-NTA resin	QIAGEN	Cat#30230
POPC, 25 mg/mL in chloroform	Avanti Polar Lipids	Cat#850457C; Cas: 26853-31-6
POPG, 25 mg/mL in chloroform	Avanti Polar Lipids	Cat#840457C; Cas: 268550-95-4
NBD-PC, 1 mg/mL in chloroform	Avanti Polar Lipids	Cat#810122C; Cas: 148533-26-0
MOPS	Sigma-Aldrich	Cat#M1254; Cas: 1132-61-2
KCl	Sigma-Aldrich	Cat#P9333; Cas: 7447-40-7
KOH	Sigma-Aldrich	Cat#221473; Cas: 1310-58-3
DMSO	Sigma-Aldrich	Cat#D8418; Cas: 67-68-5
EGS (ethylene glycol bis(succinimidyl succinate))	Thermo Fisher Scientific	Cat#21565; Cas: 70539-42-3
DSP (dithiobis(succinimidyl propionate))	Thermo Fisher Scientific	Cat#22586 ; Cas: 57757-57-0
Bovine serum albumin, fatty acid free	Sigma-Aldrich	Cat#126609; Cas: 9048-46-8
Dithionite (sodium hydrosulfite)	Sigma-Aldrich	Cat#157953; Cas: 7775-14-6
HEPES	Sigma-Aldrich	Cat#H3375; Cas: 7365-45-9

**Recombinant DNA**		

pQE9 6xHis-mVDAC1 WT	Queralt-Martín et al.^2^	N/A
pCold 6xHis-hVDAC1 WT	Jahn et al.^3^	N/A

**Software and algorithms**		

GraphPad Prism	N/A	https://www.graphpad.com/

**Other**		

1.2 mL cryogenic vial	Corning	Cat#430487
Kimble Kontes Glass Dounce homogenizer, 40 mL	VWR	Cat#885300-0040
40 mL chromatography column	DWK Life Sciences	Cat#420401-1520
Amicon Ultra 15 mL centrifugal filter, 10K MWCO	MilliporeSigma	Cat#9010
Amicon Ultra 15 mL centrifugal filter, 50K MWCO	MilliporeSigma	Cat#9050
Superdex 200 increase 10/300 GL	Cytiva	Cat#28990944
0.22 μm S-Pak filter	MilliporeSigma	Cat#GSWG047S6
0.2 μm Acrodisc syringe filter	Cytiva	Cat#4612
TLA 100.3 fixed-angle rotor	Beckman Coulter	Cat#349481
13 × 51 mm thick-wall polycarbonate ultracentrifuge tubes	Beckman Coulter	Cat#349622
Dialysis tubing 8-10 kDa MWCO	Thermo Fisher Scientific	Cat#08-607-065
Micro BCA Protein Assay Kit	Thermo Fisher Scientific	Cat#23235
0.7 mL spin desalting column	Thermo Fisher Scientific	Cat#89849
2 mL spin desalting column	Thermo Fisher Scientific	Cat#89889
250 μL Hamilton 700 series syringe	Hamilton	Cat#80765
1 mL Hamilton 1000 series syringe	Hamilton	Cat#81365
Rotavapor	Büchi	Model: R-215
Vacuum controller	Büchi	Model: V-850
Vacuum pump	Büchi	Model: V-700
Heating bath	Büchi	Model: B-491
High-pressure liposome extruder	Genizer	Model: Gextruder-10mL; Cat#GJE-10mL
Whatman Polyester Drain Disc, 25 mm	Cytiva	Cat#230600
Whatman Nuclepore track-etched membrane, 25 mm diam., 0.4 μm pore size	Sigma-Aldrich	Cat#WHA10417106
Whatman Nuclepore track-etched membrane, 25 mm diam., 0.2 μm pore size	Sigma-Aldrich	Cat#WHA10417006
Bio-Beads SM-2 adsorbent media	Bio-Rad	Cat#152-3920
FluoroMax spectrofluorometer	Horiba Scientific	Model: FLUOROMAX_PLUS_C
Polystyrene fluorimeter cuvettes	Sigma-Aldrich	Cat#C0918

## Materials and equipment

• 30% w/v LDAO stock solution: dissolve 3 g LDAO in 8 mL ddH_2_O. Make up to 10 mL with ddH_2_O.
○ Store LDAO powder and 30% solution at −20°C.

• IPTG stock solution: dissolve 1.2 g IPTG in 5 mL ddH_2_O.
○ Filter sterilize through 0.2 μm syringe filter and store in 500 μL aliquots for several months at −20°C.

• 0.5 M EDTA: Add 8.0 mL ddH_2_O to 1.46 g EDTA and adjust pH to 8.0 with NaOH (∼200 mg of NaOH pellets or ∼0.5 mL 10 M NaOH solution). EDTA will not dissolve until the pH reaches ∼8.0. Top up to 10 mL with ddH_2_O.
○ Store for several months at 4°C.

**Table:** Resuspension Buffer

Reagent	Final concentration	Amount
Tris-HCl, pH 8.0 (1 M)	50 mM	25 mL
EDTA, pH 8.0 (0.5 M)	2 mM	2 mL
Sucrose	20% (w/v)	100 g
ddH_2_O	N/A	Bring up to 500 mL
**Total**	**N/A**	**500 mL**

Filter sterilize. Do not autoclave solutions containing sucrose. Store at 4°C for several months.

**Table:** Inclusion Bodies Wash Buffer

Reagent	Final concentration	Amount
Tris-HCl, pH 8.0 (1 M)	20 mM	10 mL
NaCl (5 M)	300 mM	30 mL
CaCl_2_ (1 M)	2 mM	1 mL
ddH_2_O	N/A	Bring up to 500 mL
**Total**	**N/A**	**500 mL**

Filter sterilize or autoclave. Store at 20°C–24°C for immediate use or 4°C for several months.

**Table:** Equilibration Buffer

Reagent	Final concentration	Amount
Tris-HCl, pH 8.0 (1 M)	20 mM	10 mL
NaCl (5 M)	300 mM	30 mL
Urea	8 M	240 g
ddH_2_O	N/A	Bring up to 500 mL
**Total**	**N/A**	**500 mL**

Combine Tris, NaCl, and water to 300 mL. Add 50–100 g urea at a time until all urea dissolves. Make up to 500 mL. Filter sterilize. Store at 20°C–24°C for immediate use or 4°C for up to a month.

• 5 M imidazole: dissolve 3.4 g imidazole in 8 mL ddH_2_O. Adjust to pH 8.0 with HCl (∼500 μL 12 N) and top up to 10 mL with ddH_2_O.

Store at 4°C protected from light for several months.

• Ni-NTA Wash Buffer (20 mM imidazole): Add 600 μL 5 M imidazole to 150 mL Equilibration Buffer.

Prepare fresh buffer.

• Ni-NTA Elution Buffer (150 mM imidazole): Add 4.5 mL 5 M imidazole to 150 mL Equilibration Buffer.

Prepare fresh buffer.

• 1 M DTT: Dissolve 772 mg DTT in 5 mL ddH_2_O.

Store in aliquots at −20°C for several months.

**Table:** Rapid Dilution Refolding Buffer

Reagent	Final concentration	Amount
Tris-HCl, pH 8.0 (1 M)	20 mM	0.2 mL
NaCl (5 M)	150 mM	0.3 mL
LDAO (30%, w/v)	1.5% (w/v)	0.5 mL
DTT (1 M)	10 mM	0.1 mL
ddH_2_O	N/A	7.9 mL
**Total**	**N/A**	**9 mL**

Prepare fresh buffer. Cool to 4°C before use.

Final concentrations are calculated for a 10 mL final volume, after the addition of 1 mL purified VDAC1 in step 18.

**Table:** Dialysis Buffer

Reagent	Final concentration	Amount
Tris-HCl, pH 8.0 (1 M)	20 mM	20 mL
NaCl (5 M)	150 mM	30 mL
DTT (1 M)	1 mM	1 mL
ddH_2_O	N/A	949 mL
**Total**	**N/A**	**1 L**

Filter sterilize or autoclave before adding DTT. Buffer can be stored at 4°C for several months without DTT. Add DTT just before use in step 21. Cool to 4°C before use.

**Table:** Size Exclusion Chromatography (SEC) Buffer

Reagent	Final concentration	Amount
Tris-HCl, pH 8.0 (1 M)	20 mM	5 mL
NaCl (5 M)	150 mM	7.5 mL
LDAO (30%)	0.1%	0.83 mL
ddH_2_O	N/A	Bring up to 250 mL
**Total**	**N/A**	**250 mL**

Filter sterilize through 0.22 μm membrane and de-gas under vacuum for 4 h. Degassing can be reduced to 15–30 min if buffer is sonicated in a bath sonicator while under vacuum. Store at 4°C for 1–2 months. De-gas again before use after longer periods of storage (several months).

**Table:** Reconstitution Buffer

Reagent	Final concentration	Amount
MOPS-KOH, pH 7 (100 mM)	10 mM	5 mL
KCl (1 M)	100 mM	5 mL
ddH_2_O	N/A	40 mL
**Total**	**N/A**	**50 mL**

Prepare from filtered or autoclaved stock solutions. Store at 20°C–24°C.

**Table:** Crosslinking Buffer

Reagent	Final concentration	Amount
MOPS-KOH, pH 7 (100 mM)	10 mM	5 mL
KCl (1 M)	100 mM	5 mL
LDAO (30%)	0.05%	83 uL
ddH_2_O	N/A	40 mL
**Total**	**N/A**	**50 mL**

Prepare from filtered or autoclaved stock solutions. Store at 20°C–24°C.

**Table:** Activity Assay Buffer

Reagent	Final concentration	Amount
HEPES-NaOH, pH 7.4 (500 mM)	50 mM	5 mL
NaCl (5 M)	150 mM	1.5 mL
ddH_2_O	N/A	43.5 mL
**Total**	**N/A**	**50 mL**

Prepare from filtered or autoclaved stock solutions. Store at 20°C–24°C.

**CRITICAL:** EDTA, DTT, and detergents in powdered form (SDS and LDAO) can irritate the skin, eyes, and the respiratory system. Make sure to wear PPE when handling these chemicals, and thoroughly wash any exposed areas.

***Alternatives:*** We recommend use of a high-pressure extruder for processing large volumes of liposomes. We list the Genizer Gextruder-10 mL in the key resources table. The LIPEX Flow Model 10 mL is a good alternative high-pressure extruder. We recommend the Avanti Polar Lipids mini-extruder (Cat#610000) as a cheaper alternative and for processing smaller volumes (up to 1 mL).

## Step-by-step method details

### Protein production

**Timing: 2 days**

Figure 1 provides a graphical overview of the workflow for this step. Cells transformed with 6xHis-hVDAC1 or 6xHis-mVDAC1 expression plasmids are cultured to exponential phase growth. VDAC1 expression is induced with isopropyl β-D-1-thiogalactopyranoside (IPTG), a non-metabolizable structural analogue of allolactose. Cells are then harvested by centrifugation and either stored at −80°C or used immediately for protein purification. 1 L of culture typically yields about 5 g of cells, 50 mg of inclusion bodies, and 5 mg of purified protein.

**CRITICAL:** Sterile technique must be used when preparing bacterial cultures. Use sterile pipet tips and wipe down benchtops and equipment with 70% ethanol.

1. Start a small “overnight culture” in the evening.
  a. Transfer 50 mL sterile LB to a 250 mL Erlenmeyer flask.
  b. Add the proper selective drugs.
    i. pCold 6xHis-hVDAC1: 50 μL 100 mg/mL ampicillin (final 100 μg/mL ampicillin).
    ii. pQE9 6xHis-mVDAC1: 50 μL 100 mg/mL ampicillin and 50 μL 50 mg/mL kanamycin (final 100 μg/mL ampicillin and 50 μg/mL kanamycin).
  c. Inoculate the liquid media with a single colony of transformed *E. coli* from an LB-agar selection plate (see before you begin: transform E. coli with VDAC1 expression plasmid).
2. Grow 16 h at 37°C, shaking at 250 rpm (OD_600_ ≈ 10).
3. The next morning, add the proper selective drugs to two 2L Erlenmeyer flasks, each containing 500ml sterile LB.
  a. pCold 6xHis-hVDAC1: 500 μL 100 mg/mL ampicillin (final 100 μg/mL ampicillin).
  b. pQE9 6xHis-mVDAC1: 500 μL 100 mg/mL ampicillin and 500 μL 50 mg/mL kanamycin (final 100 μg/mL ampicillin and 50 μg/mL kanamycin).

**CRITICAL:** Cultures should not exceed 1/4 of the volume of the flask in which they are grown (i.e., 500 mL of media in a 2 L flask, or 1 L of media in a 4 L flask).

4. Add 10 mL from the “overnight culture” to each flask. Grow at 37°C, shaking at 250 rpm, until the large cultures reach an OD_600_ ≈ 0.8–1.0 (about 2 h).
  a. Blank the spectrophotometer at 600 nm with fresh LB medium in a plastic cuvette.
  b. Using sterile technique and a sterile (preferably individually wrapped) serological pipet, transfer some culture to a plastic cuvette and record the absorbance at 600 nm.
  c. Do not return the sampled culture back to the main flask.
  d. So as not to grow past the recommended OD_600_, check culture OD_600_ after first 1 h of growth and every 15–30 min as needed after that.

***Note:*** Glycerol stocks may be prepared from the remaining “overnight culture.” Combine 400 μL of culture with 500 μL of sterilized 50% (v/v) glycerol in a screw-cap cryogenic tube. Vortex. Flash freeze in liquid nitrogen and store at −80°C. For future protein preparations, glycerol stocks should be streaked out on LB-agar plates containing the proper selective drugs, and single colonies from these plates can be used to inoculate “overnight cultures.”

5. Use sterile technique to collect 250 μL of culture for SDS-PAGE analysis of the culture before induction.
  a. Pellet cells in a microfuge tube for 5 min at 3000 × g.
  b. Discard the supernatant and resuspend cells in 10 μL 1X SDS-PAGE loading buffer.
  c. Store at 20°C–24°C until step 15 (Table 1, "uninduced").

**Table 1:** Fractions to save for SDS-PAGE analysis of protein purification

	Name	Volume loaded	Description of sample (total volume)	Step in protocol
1	Uninduced	See step 5	Total protein content of culture before inducing expression of mVDAC1 with IPTG (1 L).	5
2	Induced	See step 9	Total protein content of culture after inducing expression of mVDAC1 with IPTG (1 L).	9
3	Supernatant	3 μL	Supernatant of centrifuged cell lysate (30 mL). Should contain very little VDAC1, as most of the protein was pelleted as inclusion bodies.	13.f
4	Load	3 μL	Total soluble material to be loaded on Ni-NTA column (30 mL).	13.m
5	Flow through	3 uL	Material that did not bind to the Ni-NTA resin (30 mL).	14.f
6	Wash	3 μL	Material that was removed from Ni-NTA resin by washing with 20 mM imidazole (50 mL).	14.h
7	Elution 1	3 μL	First fraction eluted from Ni-NTA resin with 150 mM imidazole (5 mL).	14.i
8	Elution 2	3 uL	Second fraction eluted from Ni-NTA resin with 150 mM imidazole (5 mL).	14.i
9	Elution 3	3 μL	Third fraction eluted from Ni-NTA resin with 150 mM imidazole (5 mL).	14.i
6. If culturing pCold 6xHis-hVDAC1 in BL21, cool cultures in a cold room or 4°C refrigerator for 30 min. Skip this step for pQE9 6xHis-mVDAC1.
7. Add 500 μL 1 M IPTG to each 500 mL culture (final 1 mM IPTG concentration).
8. Continue shaking at 250 rpm for 16–20 h at 15°C (pCold 6xHis-hVDAC1) or for 4 h at 37°C (pQE9 6xHis-mVDAC1).
9. Collect 250 μL of culture for SDS-PAGE analysis of the culture after induction. Repeat steps 5(a)-(c) for these induced cells (Table 1, "induced").
10. Harvest the large cultures by centrifugation at 6000 × *g* for 15 min.
11. Discard the supernatant and resuspend pelleted cells in 25 mL of Resuspension Buffer per 1 L of culture.
  a. Vortex to loosen cell pellet, then transfer to a 40 mL glass homogenizer.
  b. Resuspend the pellet using 5–10 strokes with a loose-fitting pestle.
12. Transfer the resuspended cells to a plastic 50 mL conical tube.

**Pause point:** Cells may be stored at −20°C for several months before proceeding without negatively affecting yield.

### Protein purification

**Timing: ½ day (for step 13)**

**Timing: ½ day (for step 14)**

Figure 2 provides a graphical overview of the workflow for this step. Cells are lysed to harvest the inclusion bodies. Inclusion bodies are washed once and then denatured in 8 M urea to solubilize the proteins. Solubilized, denatured His-tagged VDAC1 is purified by Ni-NTA affinity chromatography and can be stored at −20°C or immediately refolded for structural and functional analysis.

**CRITICAL:** In the following purification steps, VDAC1 is either aggregated (as inclusion bodies) or denatured. Therefore, these steps can be conducted at 20°C–24°C. Once VDAC1 has been refolded (see “protein refolding”), everything must be performed at 4°C with ice-cold buffers.

**Figure 3:**
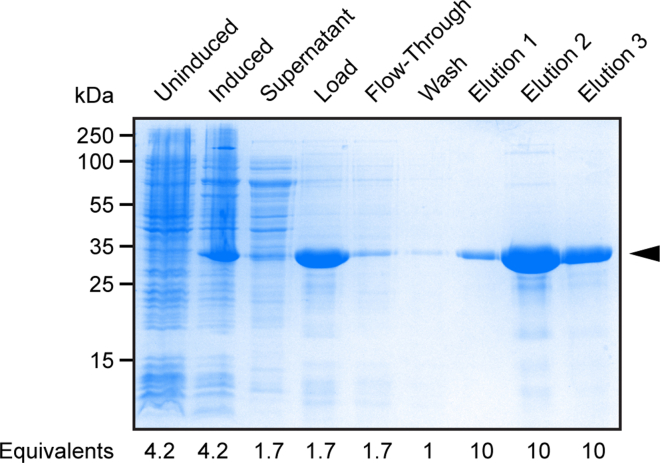
Coomassie-stained SDS-PAGE of samples collected at each stage of the purification process Arrow indicates 6xHis-mVDAC1; the relative proportion of each sample (loaded volume ÷ total sample volume, normalized to the value for the Wash sample) is indicated below the gel. To facilitate direct readout of protein recovery in each lane, we alternatively suggest loading 1/1000th of each fraction.

***Note:*** Suspensions of uninduced (step 5) and induced (step 9) cells may have become too viscous to load directly onto the gel. Brief sonication in a bath sonicator can help to reduce viscosity and facilitate sample loading.

13. Isolate and denature inclusion bodies.
  a. Thaw cells at 20°C–24°C if they were stored at −20°C.
  b. Add 3–30 mg lysozyme to the cell suspension for a final concentration of 0.1–1.0 mg/mL. Incubate for 10 min at 20°C–24°C on a rotating or rocking shaker.
  c. Add Triton X-100 to a final concentration of 0.6% (v/v; 1.5 mL 10% (v/v) TX-100). Incubate for 10 min at 20°C–24°C on a rotating or rocking shaker.
  d. Sonicate.
    i. Place sample on ice in a plastic beaker or foam cooler.
    ii. Sonicate using a probe sonicator for 3 min at 50% amplitude, 50% duty cycle.
    iii. Rest on ice for 1 min.
    iv. Repeat d(ii) and (iii) three times for a total of 9 min of sonication (see note below about total sonication time).
      **CRITICAL:** Do not let the sample overheat or foam excessively during sonication. Never sonicate glass tubes or beakers. Always wear ear protection when sonicating.
      ***Note:*** The duty cycle parameter indicates the fraction of one sonication period during which the sonicator is active or “on.” 50% duty cycle means that the sonicator will pulse on and off where the length of each “on” and “off” pulse will be the same. This means that over a total of 9 min of sonication, the sonicator will only actually spend 4.5 min on. Some machines differ in how they count the accumulated sonication time. If your machine only counts while the sonicator is on (and stops counting when it is resting), then reduce the sonication time in 14(b) to 1.5 min.
  e. Centrifuge the cell lysate at 12,000 × *g* for 15 min at 20°C–24°C.
  f. Keep 3 μL of the supernatant for SDS-PAGE analysis (Table 1 “Supernatant”).
  g. Discard the rest of the supernatant and resuspend the large white pellet of inclusion bodies in 30 mL of Inclusion Bodies Wash Buffer.
  h. Vortex to loosen pellet. Transfer the sample to a 40 mL glass homogenizer and break up any clumps with at least 10 strokes of a loose-fitting pestle.
    ***Note:*** Pelleted inclusion bodies can be difficult to resuspend and might require more than 10 strokes of the homogenizer. The pellet must be completely homogenized before solubilization.
  i. Re-pellet the inclusion bodies by centrifugation at 12,000 × *g* for 15 min at 20°C–24°C.
  j. Discard the supernatant and resuspend the pellet in 30 mL of Equilibration Buffer by vortex, followed by at least 10 strokes with a glass homogenizer.
  k. Transfer the homogenized sample back to a centrifuge tube and solubilize the inclusion bodies in Equilibration Buffer for at least 1 h at 20°C–24°C on a rotating or rocking shaker.
    **Pause point:** The solubilization step can be extended to 16–20 h at 20°C–24°C.
  l. Remove insoluble material by centrifugation at 25,000 × *g* for 45 min at 20°C–24°C.
    ***Note:*** You can begin preparing the Ni-NTA resin (step 14) during the 45-min centrifugation.
    ***Note:*** Equilibration Buffer contains 8 M urea, which denatures VDAC1 inclusion bodies and renders them soluble in solution. If the inclusion bodies were successfully solubilized, the pellet of insoluble material will be much smaller than the inclusion body pellet from step 13.g.
  m. Keep 3 μL of the supernatant for SDS-PAGE analysis (Table 1 “Load”) and save the rest for Ni-NTA affinity chromatography.
14. Nickel affinity purification of denatured protein.
  a. Prepare Ni-NTA resin.
    i. Transfer 10 mL bed volume of Ni-NTA resin to ≥ 40 mL gravity flow chromatography column. Either glass or plastic may be used.
    ii. Wash the resin with 5 column volumes (CV; 50 mL) ddH_2_O.
    iii. Wash with 5 CV Equilibration Buffer.
  b. Add 120 μL of 5 M imidazole to the solubilized inclusion bodies (supernatant from step 13.l; final 20 mM imidazole concentration).
  c. Cap the bottom of the gravity flow column and transfer the solubilized inclusion bodies to the equilibrated Ni-NTA resin in the column. Cap the top of the column. Test the watertightness of your column before use. Wrap both ends with Parafilm if necessary to prevent loss of sample due to leaks.
  d. Incubate for 1 h at 20°C–24°C on a rotating or rocking shaker.
  e. Uncap both ends of the column and collect the flow-through in a 50 mL conical tube.
  f. Save 3 μL for SDS-PAGE analysis (Table 1 “Flow Through”).
  g. Wash the column with Ni-NTA Wash Buffer (20 mM imidazole) until the absorbance at 280 nm (A_280_) reaches baseline (stops decreasing and is approximately 0).
  h. Collect the wash in a 50 mL conical tube. Save 3 μL for SDS-PAGE analysis (Table 1 “Wash”).
    ***Note:*** A_280_ usually reaches baseline within ∼5 CV.
  i. Elute VDAC1 with 3–5 CV Ni-NTA Elution Buffer (150 mM imidazole).
    i. Collect 5 mL fractions.
    ii. Measure A_280_ of each fraction in a quartz cuvette.
    iii. Continue washing until A_280_ returns to baseline (about 3–5 CV, or 6–10 fractions).
    iv. The majority of the protein usually elutes within 2 CV.
  j. Calculate the concentration of VDAC1 in each fraction using the Beer-Lambert law and absorbance at 280 nm.
    i. Blank the spectrophotometer at 280 nm with Ni-NTA Elution Buffer in a quartz cuvette.
    ii. Measure absorbance of the fractions – return the fraction to its collection tube after measurement (do not discard).
    iii. The Beer-Lambert law relates absorbance to concentration using the following equation: A_λ_ = ε_λ_bc , where A_λ_ is absorbance at wavelength λ, ε_λ_ is the molar extinction coefficient at wavelength λ, b is pathlength (usually 1 cm), and c is concentration. ε_280_ of VDAC1 = 32500 M^−1^ cm^−1^ (experimentally determined using the Micro BCA Protein Assay Kit and NanoDrop spectrophotometer).
    iv. Example calculation: a sample of 6xHis-mVDAC1 with A_280_ = 1.0 (measured in a 1 cm pathlength cuvette) has concentration c = A_280_ ÷ (ε_280_ × b) = 1.0 ÷ (32500 M^−1^ cm^−1^ × 1 cm) = 31 μM.
      **CRITICAL:** Plastic and glass cuvettes absorb strongly at 280 nm. Use quartz cuvettes to quantitate protein by absorbance at 280 nm. Rinse the cuvette thoroughly with water between each sample.
15. Run an SDS-PAGE analysis of the samples in Table 1. A 10% or 12% polyacrylamide gel is suitable. Stain with Coomassie to assess the quantity and purity of protein in each sample (Figure 3).

16. Combine pure VDAC1 fractions based on the SDS-PAGE results.
17. Concentrate protein to 300 μM (∼10 mg/mL) using a 15 mL 10 kDa molecular weight cut-off (MWCO) Amicon Ultra Centrifugal Filter housed in a 50 mL plastic conical tube.
  a. Centrifuge at 4,000 × *g* at 20°C–24°C for 5 min.
  b. Pipette the sample up and down or shake the centrifugal filter to prevent protein aggregation at the bottom of the filter.
  c. Repeat centrifugation and pipetting/shaking until the desired volume is reached.
18. Store concentrated protein in 1 mL aliquots at −20°C (∼10 mg protein/aliquot). Since the protein is denatured, there is no need to add glycerol, nor to flash freeze in liquid nitrogen.

**Pause point:** Denatured VDAC1 may be stored at −20°C for several months.

***Note:*** Depending on the conditions of cell lysis and inclusion bodies isolation, the “Load” may contain many impurities. Generally, however, the “Load” is quite pure because the inclusion bodies harvested from *E. coli* are an aggregate of principally VDAC1. Nickel affinity chromatography is still useful in this case, helping to remove minor impurities and concentrate the protein.

### Protein refolding

**Timing: 1 ½ days (for steps 19–25)**

**Timing: ½ day (for steps 26–31)**

**Figure 4:**
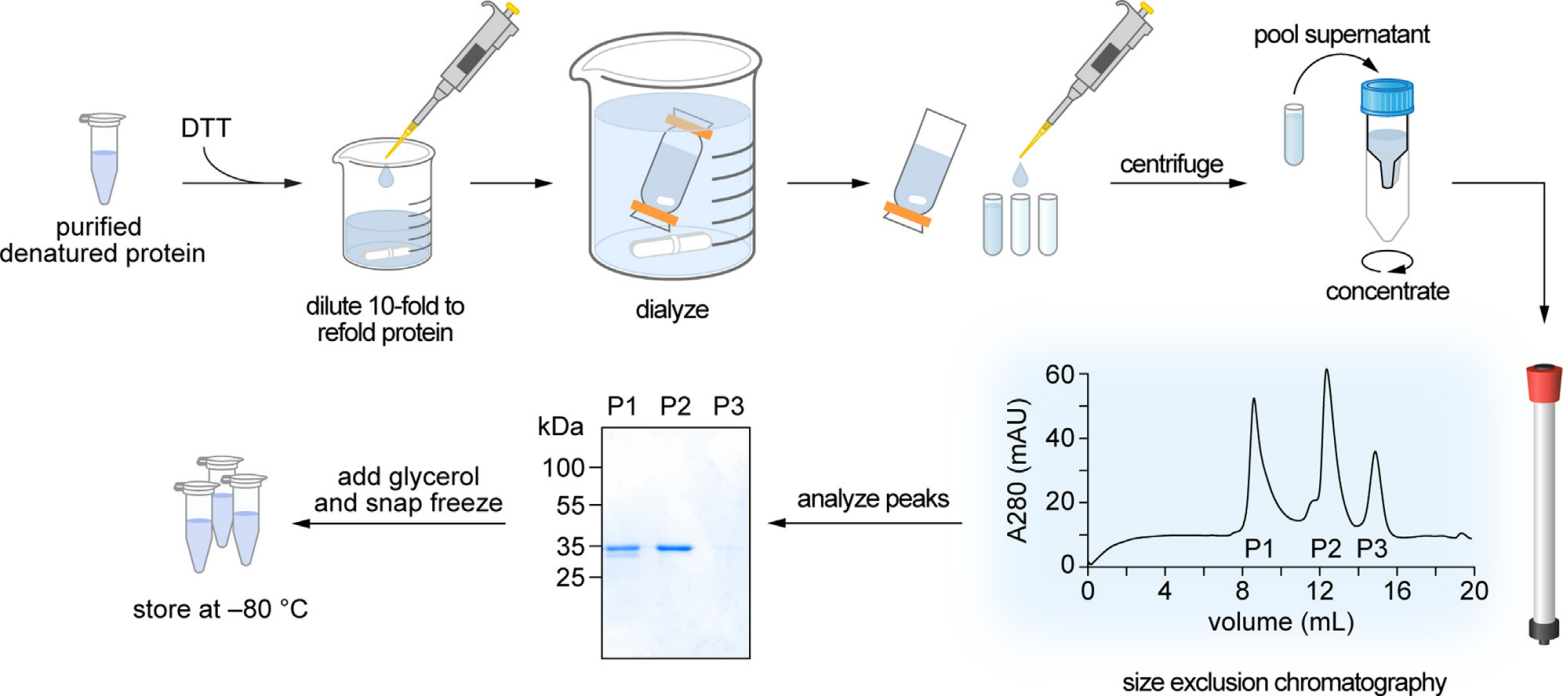
Workflow for refolding purified VDAC1 Refolding starts with diluting the purified inclusion bodies 10-fold in buffer containing the detergent LDAO. After dialysis of the diluted protein, and centrifugation to remove insoluble material and aggregates, the refolded VDAC1 is subjected to size exclusion chromatography to separate properly-folded VDAC1 monomers from multimers and any remaining aggregates.

Figure 4 provides a graphical overview of the workflow for this step. Denatured VDAC1 is refolded by rapidly diluting 10-fold to lower the urea concentration from 8 M to 0.8 M. The addition of detergent (LDAO) mimics the membrane environment to allow proper protein folding. Dialysis further decreases the urea concentration to 8 mM. The remaining urea and small aggregates or multimers are excluded from the properly folded protein by ultracentrifugation and size exclusion chromatography.

LDAO is our detergent of choice when refolding VDAC from inclusion bodies. Historically, many functional and structural studies of VDAC have been carried out in LDAO.^4^^,^^5^^,^^6^^,^^7^ N-dodecyl-β-D-maltoside (DDM) has been used to purify VDAC from membranes, but we find that refolding in DDM results in lower yields than refolding in LDAO. Additionally, we recommend using Anatrace LDAO for refolding, aswe obtain higher yields using Anatrace LDAO than other brands of LDAO.

We recommend refolding 10 mg of protein at a time at the indicated volumes and concentrations. We have found that refolding at a higher protein concentration causes aggregation and reduces the overall yield of monomeric VDAC. Some refolding protocols favor stepwise dialysis of urea rather than the rapid dilution protocol described below. We compared both methods and achieved higher yields of monomeric VDAC using the rapid dilution method.

19. If frozen, thaw 1 mL of 10 mg/mL denatured protein from step 18 at 20°C–24°C.
20. Add DTT to 2 mM (2 μL 1 M DTT) and incubate with agitation at 20°C–24°C for 30 min. This reduces all cysteine disulfide bonds to facilitate proper refolding.

**CRITICAL:** All subsequent steps should be performed on ice or at 4°C with buffers pre-chilled to 4°C.

21. Add 9 mL of cold Rapid Dilution Refolding buffer to a small beaker containing a small magnetic stir bar. While stirring, add 1 mL of denatured and reduced VDAC1 dropwise.
22. Continue stirring at 4°C all day (5–6 h).

**CRITICAL:** The quantity, quality, and reproducibility of refolded VDAC critically depend on the detergent-to-protein ratio; we suggest 15:1, w/w. Modifying this ratio may influence the multimerization of VDAC after refolding.

23. Remove the urea by Dialysis.
  a. Cut enough dialysis tubing to hold the entire 10 mL sample, leaving room for the end clips and ∼20% extra tubing to account for swelling during dialysis: for tubing with a volume/length ratio of 3.3 mL/cm, use ∼10–15 cm of tubing.
  b. Presoak the tubing in ddH_2_O for 30 min.
  c. Rinse the open tube under running ddH_2_O to remove remaining preservatives (glycerin and sodium azide).
  d. Secure one end of the tubing with a plastic clamp. Add the 10 mL protein sample from step 22 to the dialysis tubing, including the small stir bar.
  e. Secure the top with another plastic clamp. Leave a bit of air and extra tubing to allow for swelling during dialysis.
  f. Suspend the assembled dialysis sample in a 1–2 L beaker filled with 1 L cold Dialysis Buffer and a large stir bar. Remember to add DTT to the Dialysis Buffer!
  g. Gently stir at 4°C for 16–20 h. The dialysis tubing should be submerged, suspended above the bottom of the beaker but not floating on top of the buffer (Figure 2).

***Note:*** LDAO is not expected to be significantly lost during dialysis on account of its low critical micelle concentration (CMC). Thus, it is not necessary to include LDAO in the dialysis buffer.

**CRITICAL:** The volume of dialysis buffer should be at least 100 times the volume of the dialysate, so that the final concentration of urea will be ≤ 8 mM.

24. Remove the aggregated proteins.
  a. Prechill a fixed-angle ultracentrifuge rotor, such as Beckman Coulter TLA 100.3.
  b. Distribute the dialysate among four thick-walled ultracentrifuge tubes, such as Beckman Coulter 13 × 51 mm polycarbonate tubes.
  c. Ultracentrifuge at 390,000 × g for 45 min at 4°C.

**CRITICAL:** Ensure the tubes are rated for use at 390,000 × g. All tubes must be at least half full. Samples should be precisely balanced to avoid damaging the rotor and centrifuge.

***Note:*** If refolding was successful, the pellet will be very small. Mark the orientation of the tubes in the rotor as you load them in order to locate the pellet easily after centrifugation.

25. Pool the supernatant from each tube in a 15 mL 50K MWCO Centrifugal Filter. Concentrate at 4°C to less than 500 μL for size exclusion chromatography (SEC).
  a. Centrifuge the filter at 4000 × *g* in 5-min increments, as described in step 17.
  b. Centrifuge should be pre-chilled to 4°C.

***Note:*** The VDAC1-LDAO micelle complex is greater than 50 kDa, while the LDAO micelle itself is 17–22 kDa. Using a 50K MWCO Centrifugal Filter concentrates the protein without significantly increasing detergent concentration.

26. Size exclusion chromatography.
  **CRITICAL:** All buffers should be filtered through a 0.22 μm S-Pak filter, de-gassed, and pre-chilled to 4°C. Particulate matter and air bubbles can adversely affect column performance.
  ***Note:*** SEC should be run on a standard FPLC instrument (e.g., ӒKTA pure) housed in a refrigerated cabinet and equipped with a 500 μL injection loop, in-line UV monitor, and automated fraction collector.
  a. Wash the Superdex 200 Increase 10/300 GL column with 2 CV ddH_2_O. Follow manufacturer’s instructions for flow rate (0.5–0.75 mL/min) and maximum delta column pressure (2.6 MPa).
  b. Equilibrate the column with 2 CV SEC buffer.
  c. Load ≤ 500 μL of concentrated protein through a 500 μL injection loop. Monitor A_280_ and begin collecting 0.5 mL fractions after 0.3 CV (∼8 mL).
  d. Pool peak fractions based on A_280_. If more than one peak is observed (see note below and Figure 5A), pool individual peaks separately.
    ***Note:*** We usually see at least two peaks (Figures 5A and 5B): peak 1 contains small aggregates and improperly folded VDAC, peak 2 contains the monomer in an LDAO micelle, and peak 3 contains residual TX-100 micelles that may persist from cell lysis. We discard peak 1. Peak 2 has been used to obtain crystals of VDAC,^7^ and was analyzed by double electron-electron resonance,^1^ indicating that the protein in peak 2 is monomeric.

**Figure 5:**
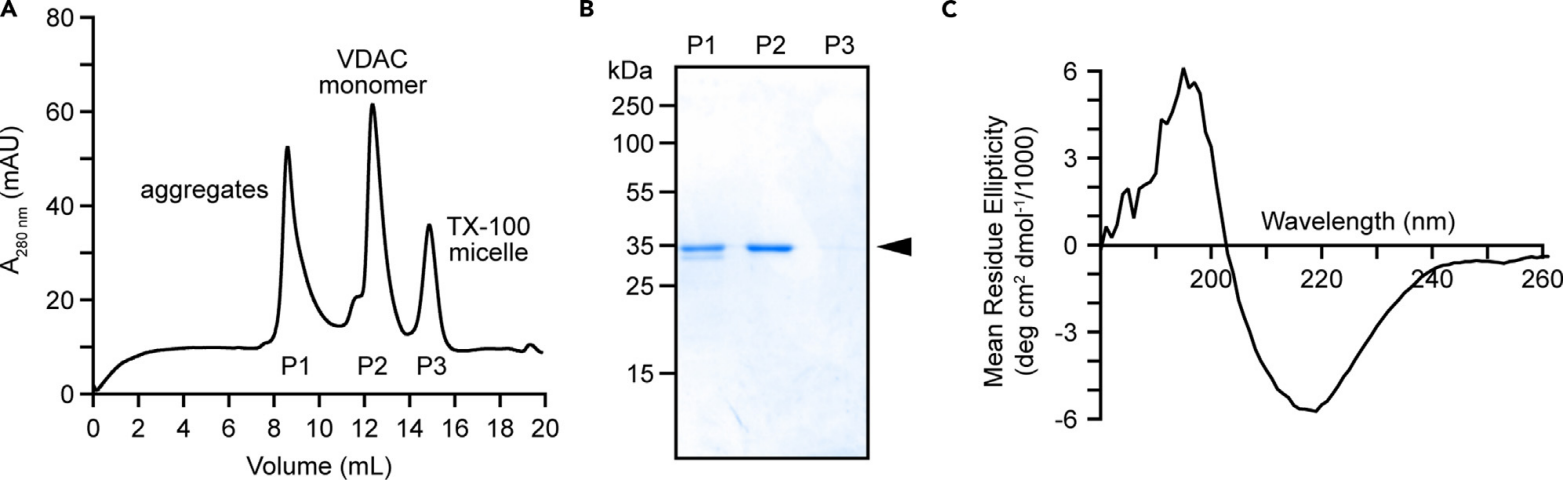
The rapid dilution method produces properly folded, monomeric mVDAC1, as assessed by size exclusion chromatography (SEC) and circular dichroism (A) SEC profile of refolded mVDAC1 collected on a Cytiva Superdex 200 Increase 10/300 GL column. Peaks 1, 2, and 3 elute at 8.6 mL, 12.4 mL, and 14.9 mL, respectively. (B) Coomassie-stained SDS-PAGE of refolded mVDAC1 eluted from the size exclusion column. Peaks 1–3 from (A) are indicated above the gel. Equivalent volumes of each peak are loaded. The faint double band observed in P1 is due to the crosslinking of the two cysteines in the improperly folded forms, leading to faster migration on the gel.^8^ (C) Circular Dichroism spectrum typical of refolded mVDAC1 showing high beta-sheet content. Data were collected on a Jasco-815 spectrometer in PBS with 0.1% LDAO.
27. Run 5 μL of each peak on SDS-PAGE and stain with Coomassie to visualize protein purity and quality (Figure 5B). Keep the peak corresponding to pure, monomeric VDAC1.
28. Quantitate protein concentration using at least two methods (e.g., A_280_, Micro BCA Protein Assay Kit, quantification of Coomassie-stained SDS-PAGE in comparison with BSA standards).
29. Based on downstream applications, VDAC1 may be concentrated using a 15 mL 50K MWCO Amicon Ultra Centrifugal Filter at 4°C.
  a. For channel and scramblase activity assays in liposomes, protein concentration must be ≥ 0.2 mg/mL.
30. For immediate use, store purified protein at 4°C.
31. Store at −80°C long-term.
  a. Add glycerol to a final concentration of 20% (v/v).
  b. Aliquot protein into microfuge tubes (we recommend 50–150 μg/tube).
  c. Flash freeze aliquots in liquid nitrogen.
  d. Store at −80°C for several months or years.

**Figure 6:**
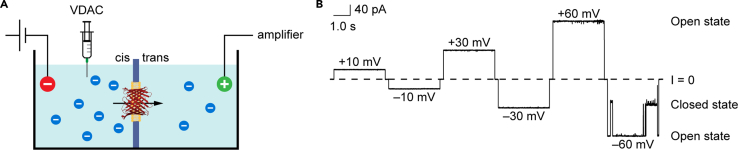
Electrophysiological measurements indicate that purified mVDAC1 is a functional channel in a lipid bilayer (A) Schematic representation of the electrophysiology setup. The setup contains two chambers separated by a Teflon film with a micrometer pore. A planar lipid membrane is formed on the pore and VDAC1 in LDAO is added to the *cis*-compartment. Current flow through the reconstituted VDAC1 channel is detected by applying potential across the membrane. (B) Representative single-channel current traces of mVDAC1 at applied voltages (± 60 mV). The dashed line indicates zero current. The recorded currents were filtered through a 500 Hz low-pass digital Bessel 8-pole filter. Characteristic open states (G = 4 nS) at low voltages and sub-conducting states (G = 2.4 nS) at higher voltages are visible. For more details on this method, see Queralt-Martìn et al.^2^

***Note:*** We typically achieve 10% yields of monomeric VDAC1 after refolding, i.e., refolding 10 mg of protein will yield 1 mg after size exclusion.

***Note:*** We validated this protocol by assessing the functionality of the protein using electrophysiology. This technique demonstrates the gating behavior of VDAC, and thus implies proper folding. We do not suggest repeating this experiment, nor do we describe it here in detail, as it requires special equipment and training. However, Figure 6 provides a schematic of the technique and typical data. For more information on this technique, see Queralt-Martín et al. (2019).^2^

### Crosslinking, reconstitution, and functional assays

**Timing: 1 day (for step 32)**

**Timing: 2 h (for step 33)**

**Timing: 1 day (for step 34)**

**Timing: 1 day (for step 35)**

**Figure 7:**
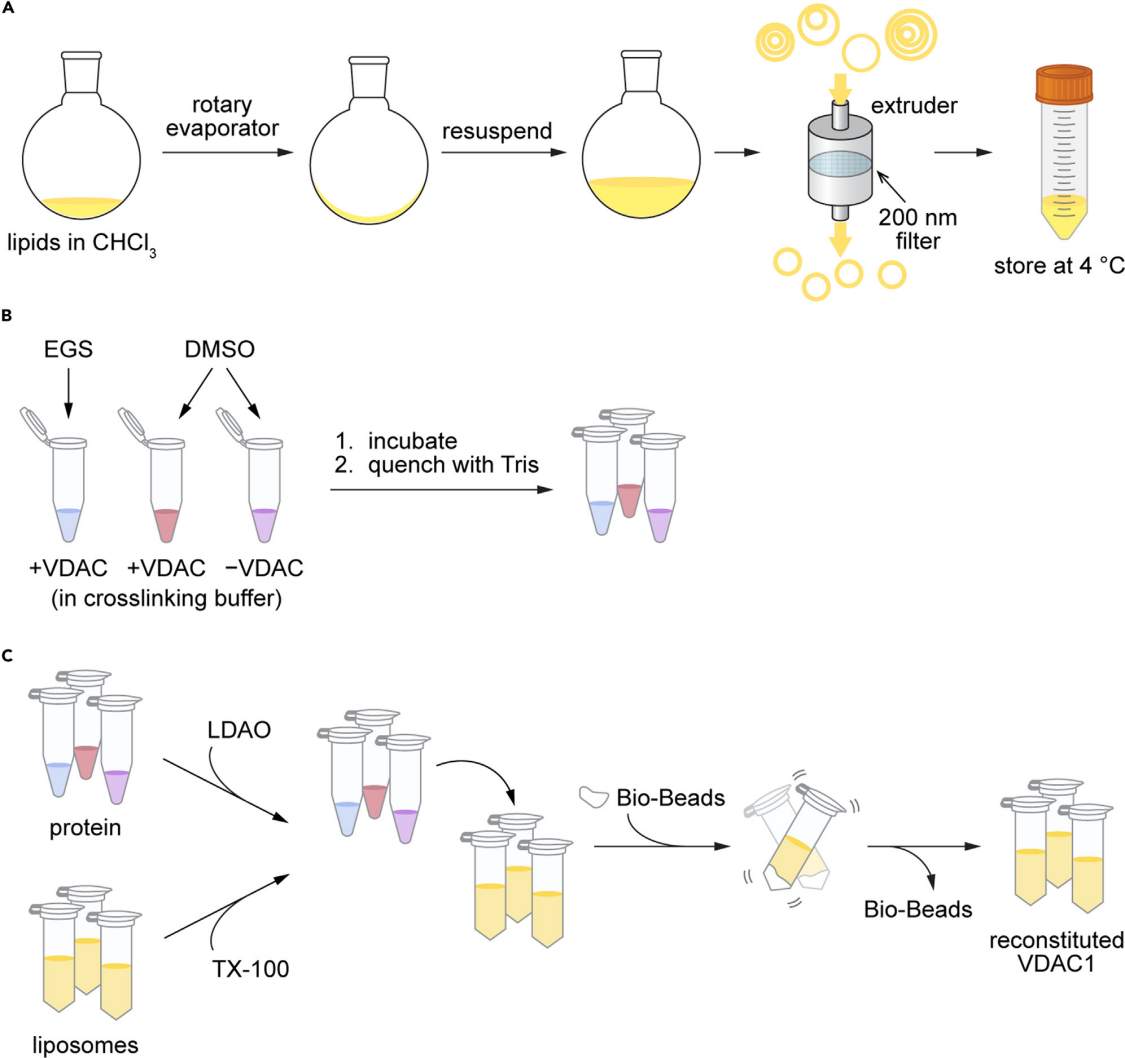
Workflow for crosslinking and reconstitution of refolded VDAC1 into membrane vesicles for functional assays (A) Lipid stocks in chloroform are dried down in a round bottom flask, resuspended in aqueous buffer, and extruded to produce unilamellar, monodisperse liposomes. (B) Purified VDAC1 is buffer-exchanged to remove Tris and crosslinked using EGS, an amino-reactive covalent crosslinker. (C) Liposomes and crosslinked (or mock-treated) protein samples are treated with detergent and incubated together for 1 h before the overnight addition of Bio-Beads. Resultant proteoliposomes reconstituted with crosslinked or mock-treated VDAC1, and liposomes containing no protein, can be tested for channel and scramblase activity via fluorescence-based assays.

Figure 7 provides a graphical overview of the workflow for this step. Large unilamellar vesicles (“liposomes”) are prepared from POPC, POPG, and fluorescent NBD-PC phospholipids. Concurrently, refolded mouse or human VDAC1 is covalently crosslinked to form scramblase-active dimers. The dimers are reconstituted into the fluorescent liposomes and assayed for channel and scramblase activity. Typically, 15 μg of VDAC1 are reconstituted into 1 mL liposomes (∼2.5 mM lipid). At this protein/phospholipid ratio, liposomes are maximally populated with active channels and scramblases, and each liposome has, on average, 30 VDAC1 dimers.

We demonstrate the reconstitution protocol using hVDAC1 (used in Jahn et al.^3^ and prepared as described above), although the same reconstitution protocol will also work for mVDAC1.

32. Liposome Preparation.
  **CRITICAL:** We recommend purchasing lipid stocks in chloroform and storing stock solutions at −20°C in glass vials or tubes with Teflon-lined screw caps. Caps should be secured with Parafilm. Chloroform is highly volatile at room temperature, and evaporation can lead to changes in lipid stock concentration. Stock vials should thus be kept on ice during use, opened only when needed to dispense the solution, and immediately returned to the freezer.
  **CRITICAL:** The NBD fluorophore is somewhat sensitive to photobleaching by ambient light. Keep all stocks and mixtures containing NBD lipids protected from light as much as possible.
  a. Clean a 100 mL round bottom flask by rinsing with water, then with methanol, and finally with chloroform. Do not clean with soap or detergent.
  b. In a fume hood, clean each Hamilton syringe by drawing chloroform into the syringe and expelling into a waste beaker. Repeat at least three times.
  c. Use the clean syringes to dispense the following volumes of lipids into the 100 mL round bottom flask: 1.45 mL POPC (36.25 mg); 160 μL POPG (4.00 mg); 195 μL NBD-PC (0.195 mg).
    ***Note:*** This mixture gives a 9:1 molar ratio of POPC:POPG and 0.5 mol % NBD-PC, with a total lipid concentration of ∼3.5 mM in 10 mL after extrusion.
  d. Using a rotary evaporator and water bath set to 37°C, dry down the lipids for 20 min at 100 rpm, maximum vacuum. The lipids should form a thin, even film on the bottom of the flask.
  e. Further dry the lipid film by placing the flask in a desiccator attached to a house vacuum line at 20°C–24°C for at least 3 h and up to 72 h. Cover the flask or desiccator with foil during this period.
  f. Add 10 mL Reconstitution Buffer to the dried lipids in the round bottom flask. Swirl and pipet up and down 20 times with a serological pipet to resuspend fully.
  g. Mix the lipids at 100 rpm for 20 min at 37°C using the rotary evaporator without engaging the vacuum.
  h. Assemble a high-pressure extruder at 20°C–24°C with a 400 nm membrane following manufacturer’s instructions.
  i. When assembling the membrane and filter supports (step 6 of manufacturer’s instructions), place one polyester drain disc followed by two 400 nm membranes on top of the stainless-steel micron pore support plate.
    ***Note:*** See “materials and equipment” for alternative extruder options (LIPEX assembly instructions can be found here). Note that for the Avanti extruder it is important to pass the sample an odd number of times through the device so that the final extrusion fills the receiving syringe rather than the one initially containing the sample (for details, see manufacturer's instructions).
  j. Pass 5–10 mL Reconstitution Buffer through the extruder.
  k. Extrude liposomes 10 times through the 400 nm membrane.
  l. Disassemble extruder, rinse with ddH_2_O, and reassemble with a 200 nm membrane.
  m. Pass 5–10 mL Reconstitution Buffer through the extruder.
  n. Extrude liposomes 4 times through the 200 nm membrane.
  o. Store liposomes in a 15 mL or 50 mL plastic conical tube at 4°C.
  p. Quantify phospholipid concentration using a colorimetric assay as described in Menon et al.^9^
    ***Note:*** After extrusion, the expected phospholipid concentration is ∼3.5 mM.
    ***Note:*** In our experience, liposomes prepared from a 9:1 molar ratio of POPC:POPG are stable at 4°C for several weeks to months, but best practice dictates using the liposomes for reconstitution within one week of preparation.
    ***Note:*** To validate the liposome preparation protocol, we measured particle size and polydispersity by Dynamic Lite Scattering (DLS) on an Anton Paar Litesizer 500 instrument. Figure 8 shows typical data from such an experiment, which are highly reproducible. Therefore, we do not recommend repeating DLS measurements as they require access to specialized equipment.

**Figure 8:**
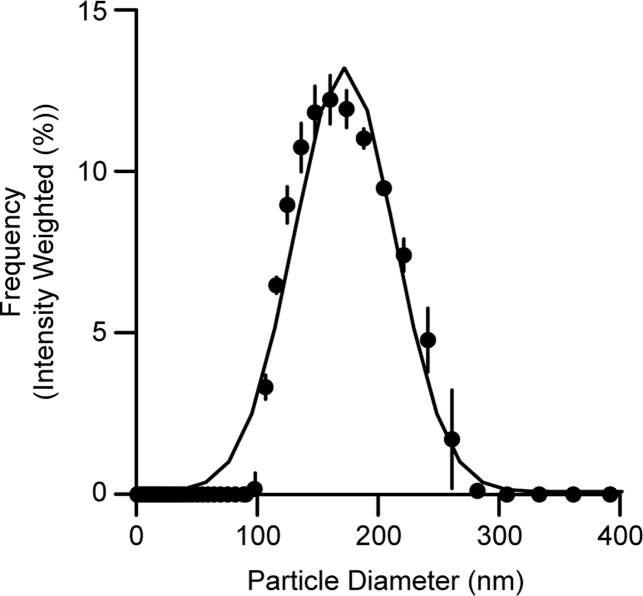
Liposome size determined by dynamic light scattering 10 μL extruded liposomes were diluted into 600 μL Activity Assay Buffer in a 1.5 mL plastic cuvette. Three independent measurements were taken on the same sample at 25°C using preset parameters for phospholipids as the material (refractive index = 1.4500, absorption index = 0.0010) and 154 mM NaCl as the solvent (refractive index = 1.3318254, viscosity = 0.000906425 Pa s, relative permittivity = 76.59715402). Error bars = mean ± SD (*n* = 3). The solid line corresponds to Gaussian least squares fit indicating a mean diameter (± SD) of 172 ± 42 nm.
33. VDAC1 Crosslinking for scramblase assay.
  ***Note:*** We describe crosslinking of refolded VDAC1 with ethylene glycol bis(succinimidyl succinate) (EGS), a non-cleavable primary amine-reactive covalent crosslinker. The same procedure can be adapted to other primary amine-reactive crosslinkers, such as dithiobis(succinimidyl propionate) (DSP), which contains a cleavable disulfide bond; we describe the cleavage protocol in step 35.d.
  **CRITICAL:** The primary amines of Tris in the SEC Buffer react with EGS to inhibit the crosslinking reaction. Therefore, VDAC1 must be buffer exchanged into a buffer without Tris or other amines before crosslinking.
  **CRITICAL:** EGS is moisture sensitive. Store desiccated at 4°C and minimize exposure of the container to air while preparing EGS stock solutions.
  a. Calculate the volume of protein needed to crosslink and reconstitute at least 36 μg of refolded VDAC1. For example, if the protein stock has a concentration of 0.5 mg/mL, buffer exchange at least 72 μL.
  b. Depending on this calculated volume, use either a 0.7 mL (30–120 μL sample) or 2 mL (200–700 μL sample) Thermo Fisher spin desalting column to buffer exchange VDAC1 from SEC Buffer into Crosslinking Buffer. Follow manufacturer’s instructions (0.7 mL column, 2 mL column) for column preparation and use.
  c. Measure new protein concentration by absorbance at 280 nm using ε = 32500 M^−1^ cm^−1^.
  d. Label three microfuge tubes for crosslinked (+EGS), mock-treated control (–EGS), and protein-free control (L) treatments.
  e. Add 18 μg of protein to +EGS and –EGS tubes. Make each tube up to 108 μL with Crosslinking Buffer. Also add 108 μL Crosslinking Buffer to tube L.
  f. Incubate all three tubes in a thermomixer at 20°C, 600 rpm, for 10 min.
  g. In the meantime, prepare 20 mM EGS and 5% DMSO.
    i. Weigh out at least 2 mg of EGS into a microfuge tube.
    ii. Add DMSO to 400 mM (5.5 μL DMSO per mg EGS). EGS will dissolve completely.
    iii. In a fresh tube, add 1 μL 400 mM EGS stock to 19 μL Crosslinking Buffer to make 20 mM EGS.
    iv. Also prepare 20 μL 5% DMSO in Crosslinking Buffer as a control.
      ***Note:*** EGS will precipitate when diluted into Crosslinking Buffer. Vortex to fully resuspend before use.
  h. Add 12 μL 20 mM EGS to +EGS tube. Add 12 μL 5% DMSO to –EGS and L tubes.
  i. Incubate in a thermomixer at 20°C, 600 rpm for 40 min.
  j. To stop the crosslinking reaction, add 1.2 μL 1 M Tris to all three tubes.
34. VDAC Reconstitution into prepared liposomes.
  a. Prepare Bio-Beads for use in step 34.i.
    i. Weigh ∼500 mg of Bio-Beads into a small (50–100 mL) beaker.
    ii. Add ∼25 mL methanol and stir gently for 15 min. Use a smooth stir bar to avoid milling the beads into a powder.
    iii. Stop stirring, let the beads settle, and drain methanol. Dispose of methanol in accordance with local and national regulations.
    iv. Repeat wash twice with fresh methanol.
    v. Wash beads twice with ∼25 mL ddH_2_O. Note that it takes longer for beads to settle in aqueous media.
    vi. Wash beads once with Reconstitution Buffer.
    vii. Discard buffer and keep Bio-Beads in fresh Reconstitution Buffer for use in step 33.e.
      ***Note:*** Keep liposomes protected from light. Opaque microfuge tubes may be used, but we prefer clear tubes wrapped in aluminum foil so that we can visually monitor the samples throughout the reconstitution procedure.
      ***Note:*** Bio-Beads may be stored in buffer for up to a week, or in water for a couple of weeks if necessary. Our practice is to wash only enough beads for the experiment at hand.
  b. To all three tubes from step 33.j, add 4.2 μL 30% LDAO (final 1% LDAO concentration).
  c. Label three additional 2 mL microfuge tubes, one each for crosslinked (+EGS), mock-treated control (–EGS), and protein-free control (L) treatments. To each tube, add 800 μL liposomes and 16 μL 10% TX-100.
  d. Incubate all six tubes (three 1.5 mL 'protein tubes' from step 34.b and three 2 mL 'liposome' tubes from step 34.c) in a thermomixer for 20 min at 37°C, 600 rpm.
  e. Into each 2 mL liposome tube, transfer 100 μL (15 μg) of protein from the corresponding 1.5 mL protein (or protein-free control) tube.
  f. Use the remaining protein to visualize crosslinking efficiency by Western Blot (step 34.p).
  g. Add 84 μL Reconstitution Buffer to each tube to make up the final volume to 1 mL.
  h. Incubate all three 2 mL microfuge tubes in a thermomixer for 1 h at 37°C, 600 rpm.
  i. Remove buffer from prepared Bio-Beads, but do not let the beads dry out. Add 140 mg Bio-Beads to each tube.
  j. Incubate in a thermomixer for 20 min at 37°C, 600 rpm.
  k. Incubate the tubes for 16 h at 4°C. We recommend an end-over-end mixer in a cold room.
  l. The next morning, gently pellet the Bio-Beads (∼700 × *g* in a tabletop centrifuge) and transfer the cleared proteoliposomes and protein-free liposomes to a fresh microfuge tube.
  m. If any particulates or Bio-Beads remain, repeat centrifugation and transfer to a fresh tube.
  n. It is advisable to do the activity assays as soon as possible, although we note that the proteoliposomes and protein-free liposomes can be stored at 4°C for several weeks without apparent detriment.
  o. Quantify phospholipid concentration using a colorimetric assay as described in Menon et al.^9^ After reconstitution, the phospholipid concentration is expected to be ∼2.5 mM.
  p. Use the remaining protein from step 34.f to visualize crosslinking efficiency by Western Blot. Run 10 μL of each sample on SDS-PAGE, transfer to nitrocellulose membrane, and detect His-tagged VDAC1 with anti-6x-His antibodies (Figure 9, dilution 1/1000).
    **CRITICAL:** DTT will cleave the disulfide bond in DSP. If crosslinking with DSP, run 10 μL of each sample on a non-reducing SDS-PAGE gel with non-reducing SDS-PAGE loading buffer and proceed with immunoblotting as usual.

**Figure 9:**
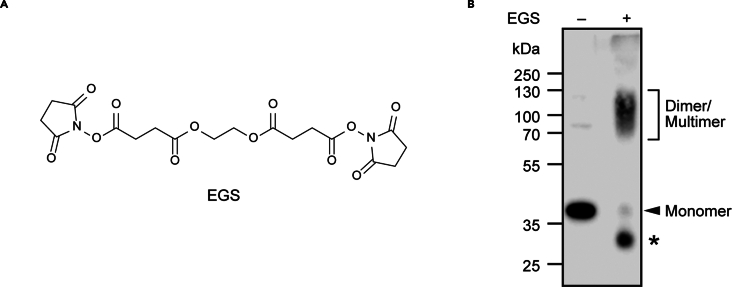
hVDAC1 dimers obtained by crosslinking with EGS (A) Chemical structure of the amine-reactive crosslinker ethylene glycol bis(succinimidyl succinate) (EGS). (B) hVDAC1 crosslinked with EGS (+EGS) or mock-treated with 5% DMSO (–EGS). Internally-crosslinked VDAC1 monomers (indicated with an asterisk) have a greater mobility on SDS-PAGE than untreated monomers because they cannot be fully denatured by SDS-PAGE loading buffer and thus maintain a more compact conformation.
35. Fluorescence-based activity assays.
  a. Prepare dithionite and BSA for channel and activity assays.
    i. Prepare at least 200 μL of fresh 75 mg/mL BSA in Activity Assay Buffer.
    ii. In three microfuge tubes, weigh out 8–20 mg of dithionite and record the mass on the tube lid.
      ***Note:*** BSA is sticky and difficult to resuspend with a pipette. We suggest adding buffer and rotating end-over-end for 5–10 min until the BSA has dissolved. Vortex and centrifuge to remove bubbles.
      **CRITICAL:** Dithionite is unstable in solution, so a fresh microfuge tube of dithionite stock should be prepared for each new measurement.
  b. Prepare two cuvettes for each proteoliposome or protein-free liposome to be assayed. Combine 2450 μL Activity Assay Buffer with 50 μL of sample and a magnetic flea for stirring. Protect from ambient light.
  c. Assay channel or scramblase activity in a temperature-controlled fluorescence spectrometer set at 20°C, set for stirring at 900 rpm.
    i. Excitation wavelength: 470 nm.
    ii. Emission wavelength: 530 nm.
    iii. Slit widths: 2.5 mm (fluorescence signal should be in the range 2–5 × 10^5^ cps).
    iv. Data acquisition frequency: 1 Hz.
    v. Allow the sample to equilibrate in the fluorimeter while collecting data. Monitor fluorescence until the signal levels off (takes about 5 min).
    vi. Abort the run and discard the data collected during equilibration.
    vii. Start a new run.
    viii. For dithionite experiments, you should now prepare one microfuge tube of 1 M dithionite by adding 0.5 M Tris, pH 10.
      ***Note:*** To prepare a 1 M (i.e., 174.1 mg/mL) solution of sodium dithionite, add a volume of Tris in μL equal to 5.7 times the mass (in mg) of pre-weighed dithionite in the tube. For example, add 57 μL of Tris to 10 mg of dithionite.
    ix. After collection data for 50 s, add 40 μL of 1 M dithionite or 40 μL of 75 mg/mL BSA through the “injection portal” using a gel-loading pipette tip.
    x. For dithionite experiments, collect 600 s of data. For BSA, collect 1000 s of data.
    xi. Save data as a dot csv file and analyze as described in “quantification and statistical analysis.”
  d. If assaying activity of VDAC1 crosslinked with a cleavable crosslinker such as DSP, treat the proteoliposomes as follows and re-assay channel and scramblase activity as in step 35.c.
    i. Prepare 1 M DTT in Reconstitution Buffer.
    ii. In a new microfuge tube, combine 100 μL of proteoliposomes containing crosslinked VDAC1 with 5 μL of 1 M DTT (final 50 mM DTT concentration).
    iii. Incubate for 30 min at 37°C, 600 rpm.

## Expected outcomes

We describe the production of monomeric VDAC1 by denaturing and refolding inclusion bodies harvested from *E. coli*. 1 L of culture is expected to yield about 5 g of cells. Figure 3 shows the successful induction of VDAC1 expression upon addition of IPTG, as evidenced by the appearance of a strong band at 35 kDa in the “Induced” lane. After washing and solubilizing the inclusion bodies, Ni-NTA purification is expected to yield 50 mg of inclusion bodies. Figure 3 shows elutions 1–3 enriched in denatured VDAC1. The rapid dilution refolding method typically results in 10% yields of monomeric VDAC from denatured inclusion bodies; following the suggestions in this protocol, refolding 10 mg at a time will yield 1 mg of monomeric VDAC1. Figure 5A shows a typical size exclusion chromatography profile for refolded VDAC1 and indicates the contents of each peak.

**Figure 10:**
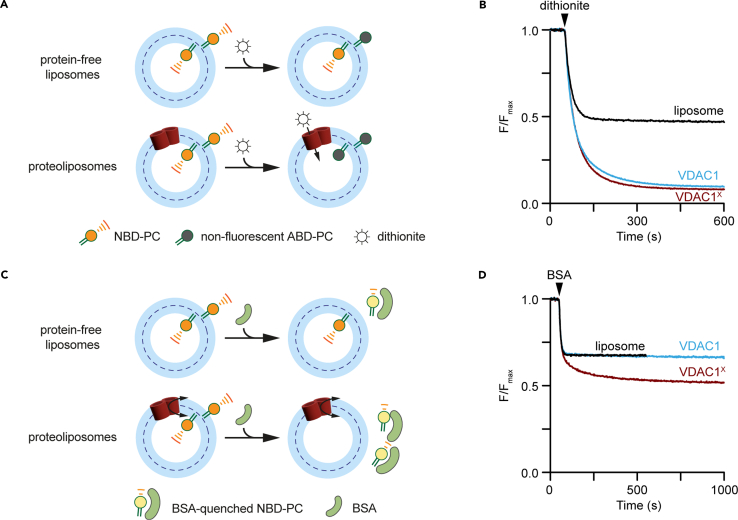
Crosslinked VDAC1 scrambles phospholipids (A) VDAC1 channel activity is assayed using the membrane-impermeant reductant dithionite. In protein-free liposomes, dithionite bleaches approximately 50% of NBD-PC fluorescence, corresponding to the phospholipids residing in the outer leaflet. In liposomes reconstituted with VDAC1, dithionite can enter the vesicles and bleach nearly 100% of NBD-PC fluorescence. (B) Time-course of NBD-PC fluorescence normalized to the average initial signal in liposomes and proteoliposomes containing crosslinked (VDAC1^X^) and mock-treated (VDAC1) hVDAC1. Vertical arrowhead indicates dithionite addition. (C) VDAC1 scramblase activity is assayed using BSA which, in contrast to dithionite, is too large to diffuse through the VDAC1 channel. BSA extracts NBD-PC from the outer leaflet and quenches its fluorescence by ∼60%, resulting in ∼30% fluorescence reduction in liposomes and ∼60 fluorescence reduction in proteoliposomes containing crosslinked VDAC1. (D) Time-course of NBD-PC fluorescence normalized to the average initial signal in liposomes and proteoliposomes containing crosslinked (VDAC1^X^) and mock-treated (VDAC1) hVDAC1. Vertical arrowhead indicates BSA addition. Liposome and VDAC1 traces overlap extensively; the liposome trace has been terminated at 500 s to facilitate visualization of the VDAC1 trace.

We describe the reconstitution of VDAC1 into fluorescent liposomes. The liposomes preparation procedure outlined here produces monodisperse vesicles with a mean diameter of ∼170 nm (Figure 8). Treatment of monomeric VDAC1 with EGS produces dimeric and multimeric VDAC1 (Figure 9). Crosslinked VDAC1 can be reconstituted into fluorescent liposomes to assay channel and scramblase activity (Figure 10); crosslinked VDAC1 is expected to be both channel and scramblase active, whereas mock-treated VDAC1 will possess comparable channel activity (Figure 10B) but scramble lipids poorly (Figure 10D).

## Quantification and statistical analysis

Fluorescence measurements are normalized to the initial fluorescence and plotted versus time. Open the data file in Excel (or comparable spreadsheet software program) and calculate the initial fluorescence value by averaging the first 50 s of data. Then normalize the data set to this average initial value.

Data are fit to a double exponential (two-phase) decay function using non-linear regression, from which rate constants and endpoint (plateau) values can be extracted. Import data into GraphPad Prism and plot normalized data versus time. Use GraphPad Prism’s built-in two-phase decay model to fit the data.

Refer to Jahn et al.^3^ methods and source files for details on data processing and sample datasets.

## Limitations

In this protocol, we describe the purification and reconstitution of mouse and human VDAC isoform 1 for functional tests and structural studies. While this protocol is readily applicable to point mutants of VDAC1, we do not describe the purification of other VDAC isoforms, though we anticipate the results will be similar.

## Troubleshooting

### Problem 1

No inclusion bodies are produced during Protein Production, resulting in a small pellet or no pellet in step 13.

### Potential solution

Either the protein was not expressed, or the inclusion bodies purification failed. SDS-PAGE analysis of samples collected at each step (Figure 3) will help to understand the problem. If no protein induction is observed in lane 2 (Figure 3), try using a fresh stock of IPTG. If the protein is expressed but not pelleted in step 13, over-sonication or excess Triton X-100 may have partially solubilized the inclusion bodies. In this case, reduce sonication time and ensure Triton X-100 concentration does not exceed 0.6%.

### Problem 2

Large pellets of aggregated or misfolded protein are observed after ultracentrifugation in step 24.c of Protein Refolding.

### Potential solution

Several factors could impact membrane protein refolding, including the quality of LDAO, buffer temperature, the ratio of sample to dialysis buffer, and dialysis timing. Make sure to use high-quality LDAO (we recommend Anatrace Sol-Grade), pre-chill buffers to 4°C, use at least 100 times more dialysis buffer than sample volume, and dialyze for at least 8 h and up to 20 h. Additionally, urea-solubilized protein can react with cyanate in solution when stored for extended periods at 20°C–24°C, leading to improper folding.^10^ Therefore, always store urea-solubilized inclusion bodies at −20°C.

### Problem 3

Phospholipid concentrations are much lower than expected — <3.5 mM after extrusion (step 32.p) or <2.5 mM after reconstitution (step 34.o) — as determined by colorimetric assay.

### Potential solution

Low phospholipid concentrations may result from loss of phospholipid during extrusion. To increase phospholipid concentration after extrusion, centrifuge extruded liposomes at 245,000 × *g* for 45 min at 4°C and resuspend in a smaller volume. It is helpful to initially resuspend the pellet in the centrifuge tube using the pestle of a small (2 mL) Dounce homogenizer. Alternatively, liposomes may be prepared at a higher concentration by using more phospholipid in step 32.c. Incubation with more Bio-Beads or for a longer amount of time than recommended (steps 34.i;k) may also contribute to loss of phospholipid.

### Problem 4

Treatment with EGS produces few or no VDAC1 dimers as detected by Western Blot (step 34.p).

### Potential solution

Tris will react with amine-reactive crosslinkers such as EGS and DSP, quenching the reaction and resulting in very low crosslinking efficiency. Make sure to buffer exchange into Crosslinking Buffer before treating with EGS or DSP. Additionally, EGS and DSP are moisture sensitive and susceptible to degradation over time. Make sure to store stock bottles desiccated at 4°C and to minimize exposure of the container to the air while preparing solutions for crosslinking. A fresh bottle of crosslinker may be required to improve crosslinking efficiency.

### Problem 5

DSP-crosslinked VDAC1 retains scramblase activity after cleavage of the crosslink with DTT (step 35.d).

### Potential solution

Running a Western Blot to visualize VDAC1 dimerization before and after treatment may help to address the issue. If VDAC1 dimers remain after DTT treatment, repeat the treatment with a fresh solution of DTT.

### Problem 6

Unexpected results are obtained during the channel and scramblase assays. For example, the extent of fluorescence reduction on adding (A) dithionite or (B) BSA to the protein-free liposome sample is less than the expected 50% and 25%–30%, respectively.

### Potential solution

(A) Dithionite is unstable in solution. Dissolve fresh dithionite before each run to ensure it is maximally active. (B) BSA may not be efficiently binding NBD-PC. Make sure to use fatty-acid free BSA.

### Problem 7

Other miscellaneous issues may arise when using the fluorimeter, such as an unstable initial fluorescence signal or erratic jumps in fluorescence.

### Potential solution

Make sure to provide enough equilibration time before adding dithionite or BSA such that the initial fluorescence signal has leveled off. This usually takes around 5 min. Additionally, make sure the stir flea is stirring at the bottom of the cuvette and not floating on top of the solution.

## Resource availability

### Lead contact

Further information and requests for resources and reagents should be directed to and will be fulfilled by the lead contact, Lucie Bergdoll (lbergdoll@imm.cnrs.fr).

### Technical contact

Questions about the technical specifics of performing the protocol should be directed to the technical contact, Lucie Bergdoll (lbergdoll@imm.cnrs.fr).

### Materials availability

Plasmids used in this study are available from the Drs. Bergdoll and Menon upon request.

### Data and code availability

Examples of original data for the channel and scramblase assays can be found in the Source files associated with Jahn et al.^3^
